# af2rave: protein ensemble generation with physics-based sampling†

**DOI:** 10.1039/d5dd00201j

**Published:** 2025-07-04

**Authors:** Da Teng, Vanessa J. Meraz, Akashnathan Aranganathan, Xinyu Gu, Pratyush Tiwary

**Affiliations:** a Institute for Physical Science and Technology, University of Maryland, College Park Maryland 20742 USA ptiwary@umd.edu; b Institute for Health Computing, University of Maryland Bethesda Maryland 20852 USA; c Department of Chemistry and Biochemistry, University of Maryland, College Park MD 20742 USA

## Abstract

We introduce 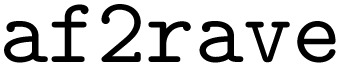, an open-source Python package that implements an improved and automated version of our previous AlphaFold2-RAVE protocol. AlphaFold2-RAVE integrates machine learning-based structure prediction with physics-driven sampling to generate alternative protein conformations efficiently. It has been well established that protein structures are not static but exist as ensembles of conformations, many of which are functionally relevant yet challenging to resolve experimentally. While deep learning models like AlphaFold2 can predict structural ensembles, they lack explicit physical validation. The Alphafold2-RAVE family of methods addresses this limitation by combining reduced multiple sequence alignment (MSA) AlphaFold2 predictions with biased or unbiased molecular dynamics (MD) simulations to efficiently explore local conformational space. Compared to our previous work, the current workflow significantly reduced the required amount of *a priori* knowledge about a system to allow the user to focus on the conformation diversity they would like to sample. This is achieved by a feature selection module to automatically pickup the important collective variables to monitor. The improved workflow was validated on multiple systems with the package 
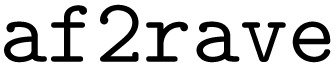
, including *E. coli* adenosine kinase (ADK) and human DDR1 kinase, successfully identifying distinct functional states with minimal prior biological knowledge. Furthermore, we demonstrate that 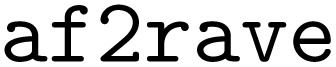 achieves conformational sampling efficiency comparable to long unbiased MD simulations on the SARS-CoV-2 spike protein receptor-binding domain while significantly reducing the computational cost. The 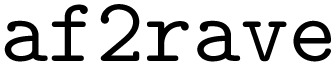 package provides a streamlined workflow for researchers to generate and analyze alternative protein conformations, offering an accessible tool for drug discovery and structural biology.

## Abstract 2

AlphaFold2 has sparked interest in generating alternative conformations for proteins. Our software suite af2rave combines AF2's predictive power with physics-based sampling to generate ensembles with vast conformational space coverage.

## Graphical abstract

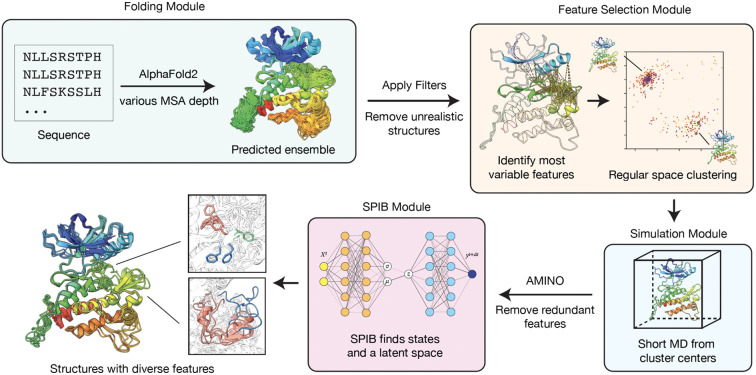

## Introduction

There is a growing consensus in protein biochemistry that protein structures should not be regarded as static snapshots of atomic coordinates but rather as ensembles of conformations.^1^ Beyond thermal fluctuations, changes in conformational preferences can happen upon many biochemical events, such as substrate binding or pH changes.^2,3^ Simply speaking, many proteins require more than one conformational state to perform their functions. Many of these “alternative” conformations are therapeutically important, as rationally designed drugs often need to target one specific state of a protein.^4^ However, structures of these metastable states are often difficult to resolve experimentally. Fast protein dynamics can exceed the time resolution of structural determination methods;^5–7^ additionally, *holo*-like structures with an empty substrate binding site are not thermodynamically preferred and hard to observe experimentally.^8^ As a result, experimentally determined structures often represent an ensemble average of all possible conformations, with a strong emphasis on the lowest free-energy “native” state.

The challenge of sampling alternative states has gained much traction with the recent surge of machine learning-based computational methods. Broadly, they can be categorized into two main groups. The first group consists of methods that train end-to-end models to directly output structures. These approaches aim to interpolate distributions of known structures to predict alternative conformations of a protein. The most notable ones in this category are those co-folding models such as AlphaFold3,^9^ its various implementations,^10,11^ RoseTTAFold All-Atom,^12^ and NeuralPlexer^13,14^ that can generate protein structures in a complex with its substrates, either another protein chain, small molecules, or nucleotides. Other ones in this category claim to directly generate alternative structures. For example, models like CFold,^15^ Distributional Graphormer (DiG),^16^ or Biomolecular Emulator (BioEmu)^17^ can directly output alternative structures, such as *holo*-like *apo* conformations. The second group of methods modifies the coevolution information input to AlphaFold2 and extracts information from them to identify alternative structures.^18–20^ Notably, it has been shown that subsampling AlphaFold2 with reduced multiple sequence alignment (MSA) depth can yield multiple states of the same protein.^21^ Several methods employ reduced MSA AlphaFold2 (rMSA AF2) as a hypothesis generator and analyze the resulting prediction ensembles to identify or enrich meaningful alternative conformations.,^22–26^ for example, using a Markov State Model.^27^

These models have gained much popularity in and outside the computational world as they can directly output structures in an end-to-end fashion requiring only a sequence as the input. However, many of those statistical models come with an inherent shortcoming: the lack of physical information.^28,29^ For co-folding models, it is an open question if they have learned actual physical interactions, or simply learned patterns in the training set, or even worse, memorized the training set.^30–32^ The challenge to incorporate physical information is also faced with these methods tweaking the MSA for structural diversity. For example, rMSA AF2 can rapidly generate a large number of conformations but does not provide information about how important or representative they are. One of the most common applications of alternative structure generation methods is in downstream drug discovery research, such as molecular docking.^33^ These tasks, especially ensemble docking, require generated alternative structures to be classified into meaningful states, such as active/inactive states or open/closed states. Structures within each state live in distinct regions of the high-dimensional protein conformation space. All of these methods fall short of telling us the topologies in this conformation space. There have been a few attempts to incorporate physical information into end-to-end models. This includes adding potential energy to the loss function^16^ or training the model with long molecular dynamics (MD) data.^17^ However, their efficacy remains to be more rigorously tested regarding (a) whether they can learn free energy information or account for entropic effects using snapshots from the protein data bank and force fields,^34^ and (b) whether the generated structures conform to the underlying Boltzmann distribution. Meanwhile, long force-field-based molecular dynamics (MD) sampling remains the gold standard for evaluating or sampling structures even in the age of machine learning.^17,35^ Other than training end-to-end models, it was also used for structural relaxation following model inference.^18^ However, it is also well known that brute force MD simulations take an astronomical amount of time to sample protein conformations “sufficiently.”^36^

Introduced originally in 2023 to sample side chain rotamers, the AlphaFold2-RAVE (
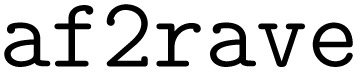
) method combines a hypothesis generator with physics-based sampling methods to address the lack of physical information in structural generation models (Fig. 4).^37^ This pipeline is built in the idea that initiating many short MD simulations from diverse initial structures should be better than waiting for one long MD simulation to cross barriers spontaneously. Thereby, it embeds AlphaFold2-generated diverse structures in a physically meaningful space, providing knowledge about which states are the more important ones and their relative relationships. AlphaFold2-RAVE starts with generating a few carefully selected rMSA AlphaFold2-generated structures that should cover more than one important conformational state. Then, short MD simulations are launched to sample the local conformational space around these centers and possibly find overlaps across the simulations. Combined, this information can provide an essential understanding of the local landscape spanned by the generated structures. The time series data from MD simulations are then analyzed using a machine learning model, State Predictive Information Bottleneck (SPIB), to uncover the underlying topology of these structures and to assign state designations to them.^38^ The RAVE protocol (Reweighted autoencoded variational Bayes for enhanced sampling) can be further used to perform enhanced sampling to explore even more in the underrepresented regions of the conformational space.^39^ Initially, AlphaFold2-RAVE was demonstrated to identify different states in proteins, ranging from sidechain rotamers to loop motions and for enhanced sampling purposes.^37,40^ Subsequent work extended its application to identifying *holo*-like structures that can be docked against and are otherwise unattainable from AF2-predicted *apo* structures.^41^

In this paper, we report an important improvement in the AlphaFold2-RAVE protocol. The AlphaFold2-RAVE protocol has been shown to be powerful in many systems.^41^ Its broader applicability is mostly hindered by two intertwining factors: namely, (a) applying the protocol often requires a large amount of prior knowledge about a system, and (b) the protocol is not user-friendly to many non-computational specialists. Researchers specialized in particular systems may not find the first one a big issue, but the second issue can be daunting for them. Computational biologists may want a more automated protocol to generate ensembles for big data analysis and would prefer it to be more automated across a large set of systems. The integrated Python package 
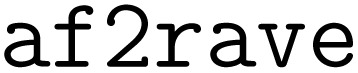
 aims to solve this problem by integrating the tools required for AlphaFold2-RAVE while providing a user-friendly interface and making it more automated. An additional feature selection module is designed to help identify important collective variables (CVs). In the original method, a set of user-defined CVs is required at the beginning of the protocol. The CVs are often distances between atoms or dihedral angles, or features to describe and distinguish the states of the system. This requires substantial knowledge about the system one wants to investigate. The new feature selection module eliminates the need to input specific CVs into the algorithm. Instead, the algorithm only asks the users to identify “key areas” of interest. A set of representative features will be automatically detected based on information acquired from AlphaFold2 and subsequent short MD simulations.

Our automated protocol and package were validated on three systems. We illustrate how this more automated pipeline—or its component modules—can be used to generate important conformational states and facilitate sampling. We selected *E. coli* adenosine kinase (ADK) and human DDR1 kinase as the first two examples. These systems have well-studied alternative states and empirical collective variables (CVs) to describe their conformational changes. In DDR1, different states are characterized by loop motions and residue rotamer shifts, whereas ADK undergoes conformational changes involving rotational motions of multiple domains. The 
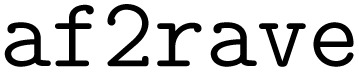
 package successfully generated meaningful alternative structures for both systems without requiring manually selected CVs. Next, we demonstrate that 
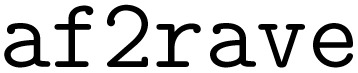
 can produce ensembles covering all relevant conformational states. This is shown for our third system, the receptor-binding domain (RBD) of the SARS-CoV-2 spike protein. By benchmarking against a published 1.8 ms MD simulation,^42^ we show that 
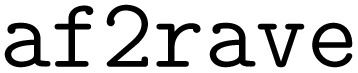
 achieves comparable coverage with just 1 μs of simulation, representing a >1000-fold improvement in efficiency.

## Results

The 
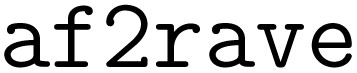
 package is a suite of Python codes published with an MIT license made publicly available at https://github.com/tiwarylab/af2rave. Two notebooks are also available in the repository to run on Google Colab. The complete pipeline can not only generate important conformations, but it also provides several useful byproducts, such as a mechanistically meaningful latent space to project structures into a two-dimensional space, making it potentially useful for enhanced sampling. In the Results section, we will focus on ensemble generation and sampling, and the properties of the latent space will be discussed in detail in the ESI.†

### Open and closed state of *E. coli* Adenosine Kinase (ADK)

**Fig. 1:**
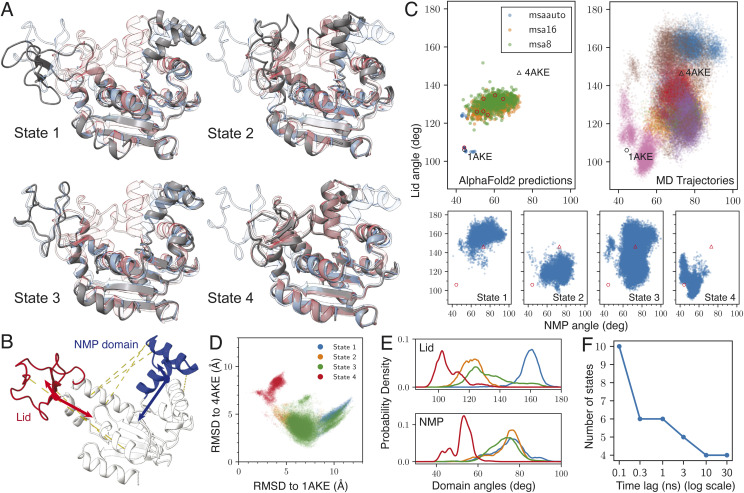
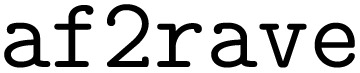
 generated structures for adenosine kinase (ADK). (A) Snapshots from the four states generated by 
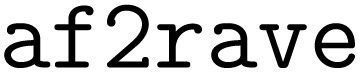
. The blue transparent structure represents the open state (PDB: 4AKE), and the red structure represents the closed state (PDB: 1AKE). The gray structure is the generated structure. (B) The lid (red), NMP binding domain (blue), and core domain (white) of ADK. The two angles are defined as illustrated. For a detailed definition, see ref. 43. The yellow dashed lines are the final 6 pairwise distances that were input to SPIB. (C) Different structures shown in the NMP angle–lid angle space. The two reference structures (1AKE and 4AKE) are represented as a hexagon and a triangle, respectively. The top left panel shows the structures generated by AlphaFold2 with three different MSA settings. The 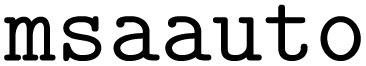 setting utilizes the most MSA information, while 
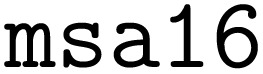
 and 
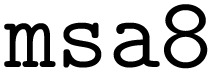
 use reduced information. Red circles indicate the 7 chosen cluster centers. The top right panel shows the MD trajectories in this space, with each color representing a single trajectory. The bottom four panels show the four states identified by SPIB. (D) The generated four states are shown according to RMSD relative to the two reference structures. (E) Histograms of the NMP and lid angles by state. The color scheme matches panel D. (F) The number of states identified by SPIB as a function of the user-tunable hyperparameter: time lag (Δ*t*).

The *E. coli* Adenosine Kinase (ADK) has two biologically relevant states. Substrate binding will trigger the protein to switch between an open and a closed state.^44^ The two moving domains are referred to as the “lid” (red) and the nucleotide monophosphate (“NMP”) binding domain (blue), which moves in a manner analogous to folding and unfolding a multitool knife, with the core domain (white) acting as the anchor point (Fig. 1B). The experimental structures of these two states are available in the Protein Data Bank, with the identifiers 1AKE (closed) and 4AKE (open), respectively.^45,46^ A commonly used method to characterize this motion is by measuring the angles between the core domain and both the lid and the NMP domain, named lid angle and NMP angle, respectively (Fig. 1B).^43^

The first step of structural generation involves generating hypotheses using reduced MSA AlphaFold2. We generated 640 structures with an MSA depth of 8 : 16, 640 structures with an MSA depth of 16 : 32, and 25 structures with full MSA, which utilizes up to 512 : 5120 sequences. These structures can cover a modest portion of the intermediate regions between the open (4AKE) and closed (1AKE) states in the lid-NMP angle space (upper left, Fig. 1C). 7 cluster centers (red circles, Fig. 1C) were picked automatically among the 1305 structures with the feature selection module. From each cluster center, we ran 100 ns MD simulations to explore the local conformational space around them. This short, 700 ns sampling effectively expanded the coverage of the Lid-NMP angle space (upper right, Fig. 1C). Using only 6 automatically selected pairwise distances as input, SPIB identified 4 states from the combined trajectories, which correspond to 4 distinct regions in the Lid-NMP angle space (Fig. 1C, lower).

Fig. 1A shows a visualization of the 4 typical structures corresponding to the 4 identified states, with 1AKE (red) and 4AKE (blue) shown in transparent colors for reference. State 3 closely resembles the open state, while state 4 is the most similar to the closed state. State 1 features a wider open lid, whereas state 2 has a closed lid but an open NMP domain. These characteristics are also evident in some statistical properties. State 4 has the lowest RMSD to 1AKE and the highest to 4AKE, whereas state 3 shows the opposite trend. States 1 and 2 are positioned adjacent to state 3 but occupy different regions in the RMSD plot (Fig. 1D). The four states exhibit distinct distributions of lid angles, while the NMP angles are similar across states 1 to 3 and for state 4 it is much smaller (Fig. 1E).

The number of states found by SPIB from the trajectory is controlled by a key hyperparameter: the time lag Δ*t*. SPIB tries to find the minimal information that is needed to predict the future states of the system after Δ*t*. Longer time lags will average out the faster dynamics of the system, leaving only the typically slower large conformation changes under the radar. Consequently, as Δ*t* increases, the number of states SPIB can identify decreases (Fig. 1F), since faster dynamics are ignored leaving larger conformational changes with slower dynamics to be recognized.^41^ In our work, we used Δ*t* = 10 ns. While the number of state changes, the quality of the state classification is agnostic of choice Δ*t* within a wide range, as it does not alter the underlying latent space structure but instead functions as a control for granularity of the labeled states. A more detailed discussion is provided in the ESI and Fig. S9.†

### A-loop and DFG motif of human DDR1 kinase

**Fig. 2:**
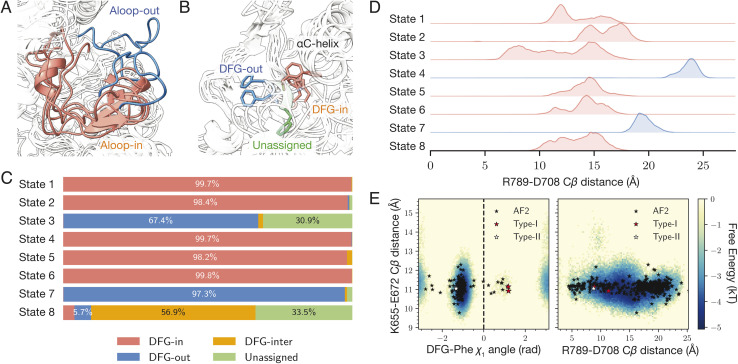
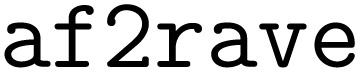
 generated structure for human DDR1 kinase. (A) and (B) Snapshots of the eight states generated by 
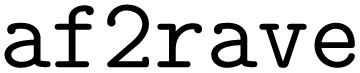
. Panel A focuses on the activation loop (A-loop), where states 4 and 7 are colored in blue (extended), and the others are colored in red (folded). Panel B zooms into the DFG-phenylalanine (DFG-Phe) conformation, where states 3 and 7 are colored in blue (DFG-out), state 8 in green (unassigned), and the others in red (DFG-in). (C) The distribution of the structures based on the Dunbrack DFG label. (D) Displays histograms of the distance between the C_β_ atoms of R789 and D708. Smaller distances correspond to a folded A-loop, while larger distances indicate an extended A-loop. (E) af2rave coverage projected on the DFG-Phe *χ*_1_ angle and the R789-D708 salt bridge against the K655-E672 salt bridge. Background was from the histogram of combined unbiased sampling. The two reference type-I inhibitor bound structures are from PDB entries 6BSD and 6BRJ.^47^ The type-II inhibitor bound structure is from 6FIO.^48^

DDR1 is a protein kinase characterized by two major conformational elements of interest. The activation loop (A-loop) is a flexible structural region responsible for the kinase's catalytic function. It can adopt either an extended, mostly commonly seen in active conformation, or a folded, inactive conformation (Fig. 2A). The N-terminal end of the A-loop is close to the ATP binding site, and the phosphorylation of the A-loop serves as a critical regulatory mechanism for kinase activation.^49^ Also at the N-terminal end of the A-loop lies a conserved Asp–Phe–Gly motif, commonly termed the DFG motif. This motif can adopt several conformations, including DFG-in, DFG-out, or an intermediate state occasionally observed called DFG-inter depending on the relative location of the aspartate and the phenylalanine residue (Fig. 2B).^50^ Active kinases must adopt the DFG-in conformation, where the aspartate residue points into the ATP-binding site to enable catalytic function. The DFG-out conformation involves rotation of the aspartate away from the active site, impairing catalysis while exposing an allosteric pocket.^51^ Drugs binding to DDR1 can target either the DFG-in state, competing with ATP, or the DFG-out state at the allosteric pocket, locking the kinase in its inactive state.^4,52,53^ The conformational state of the DFG motif is typically defined using two structural metrics known as the Dunbrack distances, which measure the distance from the terminal atom of the DFG-phenylalanine to two anchor atoms within the nearby αC helix.^50^ Conformations are classified as DFG-in, DFG-out, or DFG-inter based on these distances, while structures that fall outside these defined thresholds are labeled as “unassigned.” Similarly, the A-loop conformation can be commonly assessed by the distance between two salt bridge-forming residues R789 and D708 (using the numbering in Uniprot Q08345). For this study, we characterized the A-loop conformation using the distance between their C_β_ atoms.^41^

The 
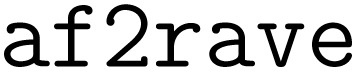
 protocol identifies 8 states of the system with distinct A-loop conformations and DFG labels (Fig. 2A and B). These eight states can collectively cover most possible combinations of DFG and A-loop conformational states. Fig. 2C provides a description of these states in terms of their respective Dunbrack DFG label distribution. States 1, 2, 4, 5, and 6 (colored in red in Fig. 2A) prominently display the DFG-in conformation with little variation towards other conformations; states 3 and 7 (colored in blue) display the DFG-out conformation with the former at a lower frequency than the latter; state 8 (colored in green) has a majority DFG-inter conformation with unassigned as the second largest distribution. The difference in A-loop conformations, characterized by distance between C_β_ atoms of the R789-D708 salt bridge, can also be distinguished among states. States 4 and 7 (colored in blue in Fig. 2A) have an extended A-loop with a larger salt bridge distance; while other states (colored in red) display a much smaller separation, indicating a folded A-loop. A few other projections also showed 
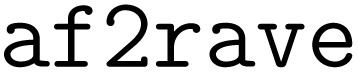
 can expand the coverage of conformational sampling. For example, all sampled conformations were projected in the space of the C_β_ distance of the conserved K655-E672 salt bridge, against both the DFG-Phe *χ*_1_ angle and the R789-D708 salt bridge C_β_ distance (Fig. 2E). Overlayed on af2rave trajectory coverage are rMSA AF2 (black stars) and three *holo* structures, respectively. These *holo* structures are either bound to a type-I inhibitor (red) or a type-II inhibitor (white).^54^ These *holo* structures lie in different basins in these projections, and 
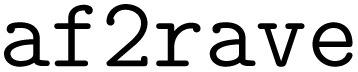
 sampling covers all of them.

### 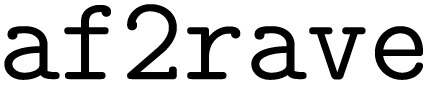 reaches similar level of sampling as millisecond long MD simulations

Another aim of the 
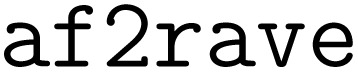
 protocol is to facilitate sampling by selecting well-chosen starting structures. Instead of relying on long MD simulations to cross free energy barriers spontaneously, starting from diverse configurations in multiple basins can be equivalent to “tunneling” through those barriers, with the goal of achieving comparable coverage of conformational space through shorter MD simulations. In this approach, AlphaFold2 serves as a hypothesis generator, while the feature selection module clusters the generated structures into diverse MD-starting configurations.

**Fig. 3:**
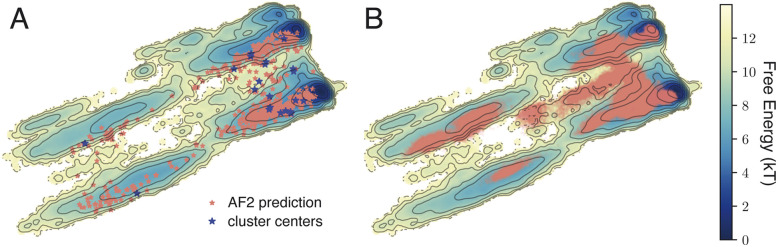
Sampling of the SARS-CoV-2 spike protein receptor-binding domain (RBD). The background topology illustrates the free energy computed from a 1.8 ms-long unbiased MD simulation. The trajectory was mapped onto a tICA space generated using the sines and cosines of backbone torsion angles. (A) AlphaFold2-predicted structures mapped onto this space, along with the selected cluster centers. These structures span all four major basins. (B) The 21 MD trajectories, each 50 ns long, mapped onto the same space.

For the SARS-CoV-2 spike protein receptor-binding domain (RBD), a 1.8 ms-long MD trajectory starting from an experimental structure (PDB: 6M0J) is publicly available.^42^ Given the availability of this long MD simulation, time-lagged independent component analysis (TICA) was a natural choice to project the structures into a 2*D* space for visualization.^16^ Using a time lag of 10 ns, we computed the free energy in this space, revealing four distinct basins (background topology in Fig. 3; see ESI Methods†).

To compare with the conventional MD approach, we applied 
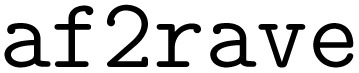
 to generate 640 structures using AlphaFold2 for the same sequence. These structures were projected onto the TICA space derived from the long MD trajectory (Fig. 3A, pink dots), and remarkably, they spanned all four basins without additional simulation. The feature selection module of 
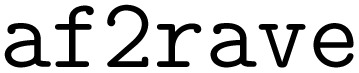
 then automatically identified 200 collective variables (CVs) from all possible C_α_ pairwise distances. To maximize conformational coverage while minimizing computational cost, we increased the number of cluster centers and reduced individual simulation lengths, maintaining a total workload of approximately 1 μs. By applying a smaller distance threshold during clustering, 21 representative configurations were selected as initial structures (Fig. 3A, blue stars). Each was simulated for 50 ns (instead of 100 ns), yielding a total simulation time of 1.05 μs.

Projecting these new trajectories onto the same TICA space (Fig. 3B) revealed that our simulation has explored all four basins. This demonstrates that diverse starting structures generated by 
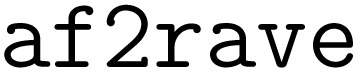
 effectively bypassed free energy barriers between conformational states, accelerating sampling compared to traditional long MD simulations. Despite using only approximately 1 μs of aggregate simulation time, the protocol achieved coverage comparable to the original 1.8 ms trajectory, capturing nearly all key regions in the TICA space.

## Discussion

In this work, we investigated three systems to show two important features of the 
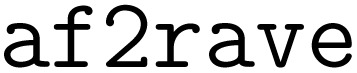
 package, structural ensemble generation and expedited sampling. We showed that with minimum prior knowledge about collective variables, our protocol can generate meaningful conformation ensembles for ADK and DDR1 and classify them into biologically relevant states. With the RBD of the SARS-CoV-2 spike protein, we showed that good sampling on the conformational landscape can be achieved by significantly shorter sampling. The success of the 
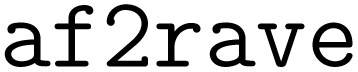
 protocol demonstrated one key idea: short simulations from wisely chosen staring points can gather enough information about the more global conformational landscape of interest. This “tunneling” strategy bypasses slow barrier-crossing events by initializing simulations in distinct basins, decoupling sampling efficiency from barrier heights. The results demonstrate that combining AI-derived structural hypotheses with adaptive MD initialization accelerates conformational landscape mapping, offering a generalizable framework for studying biomolecular systems where long-timescale sampling remains prohibitive.

Admittedly, we are not yet in an era where enough data exist to train one single end-to-end model capable of solving all structural biology challenges. The AlphaFold2-RAVE pipeline aims to take the guesswork out from those black box models and prioritizes a physically interpretable foundation over rapid inference and universality. We will then discuss some of the information we still need from the user to specify what their goal is.

One important piece of knowledge to incorporate into the workflow is the user's pick of structural features they would like to sample, which dictates the choice of MSA depth. Although a fully automated workflow sounds appealing, this requirement is absolutely necessary to ensure the conformation diversity sampled matches the expectation of the user. Protein states exist in different timescales. The switching between active and inactive states between kinases can be at the timescale of minutes,^55^ while the RBD conformation changes in the reference MD trajectory happen faster than milliseconds. Usually speaking, shallower MSA depths and thus weaker coevolution signals allow AlphaFold2 to sample conformational transitions with longer timescales. This is the rationale behind our choice of different MSA depths for our three systems. We need MSA depth 8 to sample slow activation loop movements in kinases, and deeper MSA (with all 142 available sequences) to sample faster spike protein RBD conformational movements. Shallower MSA subsampling on RBD will yield different tertiary structures from the crystal reference 6M0J, which is also the starting point of the long, reference MD. In the reference millisecond-long MD simulation, the structures along the trajectory are mostly within 5 Å RMSD from the starting structure (Fig. S5†). Many reduced MSA structures have RMSDs up to 20 Å, but can hold within 100 ns of short MD simulation (Fig. S8†). This suggests that the reduced MSA structures were too far removed from the conformational landscape explored by unbiased MD simulations. These misfolded conformations may be interesting to those who study protein denaturing, but is out of the scope for most functional studies.

Our 
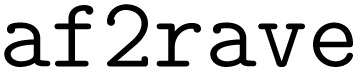
 package can automatically detect important features to look at, and this feature selection module has made our previous work much more generalizable to new systems with little prior knowledge, although information about which part of the protein one likes to sample remains an important input. For example, if one wants to sample the conformation diversity at the interface between an antibody and antigen, a selection of atoms can be input to the feature selection module to ensure the algorithm focuses on this particular region. The antigen may have some other flexible, unfolded parts, and it can be left out to reduce noise. In our previous work with DDR1, 14 pairwise distances were hand-picked based on prior knowledge of the system.^40^ In contrast, in this work, we only included a few sidechain atoms known to be associated with DFG flipping, along with all the C_α_ atoms from the activation loop and nearby regions (see the ESI†). This input resulted in 11 automatically selected pairwise distances, two of which involved the terminal ζ-carbon of the DFG-phenylalanine. These two CVs are similar, but not identical to the Dunbrack distances, which is a common CV used to monitor DFG flipping.^50^ The two CVs output by SPIB, which are linear combinations of these 11 distances, are shown to capture both A-loop movement and DFG flipping.

Performance-wise, the efficiency of the 
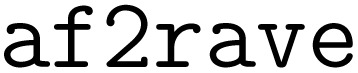
 protocol primarily depends on the efficiency of the MD simulations. Some programming is also needed to assemble the parts together. For proteins with 200 to 300 residues, the folding module requires approximately 3–10 seconds to generate one structure, depending on the MSA depth. The feature selection module does not involve computationally intensive steps. The protocol then requires short MD simulations from several cluster centers. These simulations require the most GPU hours but are highly parallelizable, depending on available resources, and typically take from a few hours to a day. Afterward, the time series data are processed using the AMINO algorithm. This step is memory-intensive but generally completes quickly. Finally, the SPIB module can finish in a few minutes for a single Δ*t*. As previously mentioned, the final SPIB latent space definition is transferable between homologs with similar tertiary structures because the input features are transferable. For example, we found that the latent space is fully transferable between DDR1, Abl1, and Src kinases.^41^ This transferability could further improve the efficiency of the protocol when working with multiple proteins within the same family.

With the recent availability of AlphaFold3 and other models,^9–11^ AlphaFold2 remains the best choice as a hypothesis generator for our task. Most importantly, AlphaFold2 allows for easier tuning of MSA depth to achieve different levels of structural diversity. The recycle and dropout parameters can also be adjusted to introduce more stochasticity into the inference process. AlphaFold3-like models emphasize less on coevolutionary data and instead use diffusion-based generative models to introduce stochasticity. Such stochasticity (noise) is often not the structural diversity (*i.e.* open and close states) we are looking for, and it is also more difficult to tune. The difference in structural generation architectures also makes AlphaFold2 significantly faster in inference compared to diffusion-based AlphaFold3.

## Methods

**Fig. 4:**
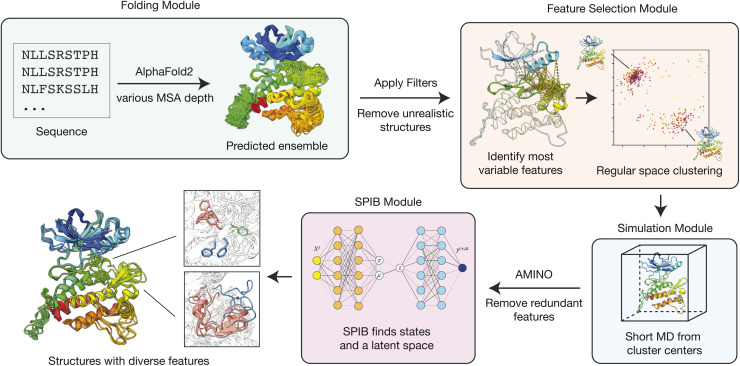
The workflow of AlphaFold2-RAVE. AlphaFold2-RAVE (
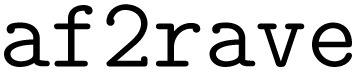
) takes a protein sequence as the input and generates diverse structures using a modified AlphaFold2 protocol with varying reduced MSA depths. The resulting structures are first passed through a filter (often by RMSD to a reference structure) and then analyzed as an ensemble to identify the most variable pairwise distances within a user-defined selection, providing a coarse set of features to work with. Subsequently, regular-space clustering selects a few representative structures as cluster centers to initiate short MD simulations. The resulting trajectories provide additional sampling to further reduce the initially selected features to a smaller set with AMINO. Finally, SPIB is applied to these reduced degrees of freedom to define states and label the structures. A 2D latent space is simultaneously generated to visualize the relative relationships between the structures, which can also be used as collective variables (CVs) for potential further enhanced sampling.

A tutorial and documentation of the code are also available in the repository. Fig. 4 shows the workflow of the 
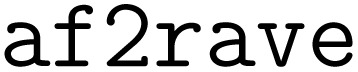
 package and it will be discussed in this section. Details about the simulation and data analysis used in this work are available in the ESI.†

### Folding module

The folding module wraps the ColabFold package to perform reduced-MSA AlphaFold2 inferences.^56^ MSA generation was performed using the ColabFold MMseqs2 web server.^57^ The user can specify the MSA depth based on their requirements. Generally speaking, a shallower MSA depth can sample conformation changes corresponding to a longer timescale but reduces structural confidence. Additionally, structures generated with different MSA depths can be combined together for subsequent analysis.

### Feature selection module

The feature selection module analyzes the structures generated by the folding module and provides (a) a few representative structures (cluster centers) to initiate MD simulations and (b) several hundred pairwise distances to monitor during MD simulations. First, the structures are passed through an RMSD filter, which by default uses the structure with the highest pLDDT score as the reference to exclude unfolded or unrealistic structures. Additional user-defined filters can also be applied. In our next step, the user selects regions of the protein to compute pairwise distances. This is the stage where *a priori* knowledge about the system can be applied. In our case with ADK and DDR1, we focused on regions known to undergo conformational changes to prioritize the most relevant domains. The pairwise distance features are ranked by their coefficient of variance (CoV = variance/mean), and the top few hundred are selected. Finally, regular space clustering is applied to the selected subspace using a distance threshold to identify a few cluster centers.

The RMSD cutoff, number of features to use, and clustering cutoffs are left to the user to adjust based on the specific system. The number of features should be sufficiently large to encompass important and diverse pairs but not excessively high, as Euclidean distances used in regular space clustering become less informative with increasing dimensionality. It is recommended to select roughly the top 5% of all features, not exceeding a few hundred. The distance cutoff used in clustering determines the number of clusters identified. Depending on computational resources available, selecting 5 to 20 cluster centers is generally recommended. A detailed discussion of parameter selection for this step is provided in the ESI.†

### Simulation module

The simulation module primarily consists of organized OpenMM APIs.^58^ The two major functions of this module are (a) generating a solvated simulation box from protein structures and (b) running the MD simulation with minimal setup. The code provides an automated way to generate a simulation box for soluble proteins. Customization such as disulfide bonds is also possible. The monitored coarse set of pairwise distances will be monitored during the simulation and output with a higher frequency than the trajectory. Approximately, 100 ns of sampling starting from the cluster centers will be enough for the purpose of 
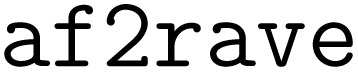
.

Inevitably, the hundreds of CVs monitored during the MD simulations will have a lot of redundancies, as they were picked only by ranking the coefficient of variance. This means if the distance between residue *m* and *n* is monitored, distances between residue *m* ± 1 and *n* ± 1 are also likely included. Handling these plentiful and redundant CVs can be challenging for subsequent analysis work. To address this, we integrated a method called Automatic Mutual Information Noise Omission (AMINO) to remove these redundant CVs.^59^ AMINO computes the mutual information across CVs using the time series data from MD simulations to identify the highly correlated one, and only keeps the most representative CVs for further analysis. This usually reduces the number of CVs from a few hundred to fewer than 20, making the following work more manageable and interpretable.

### SPIB module

The SPIB module uses the timeseries of the selected CVs as input to generate state labels and latent space representations.^38^ With this latent representation, any structure can be projected into a specific state, including those sampled by MD simulations or more crystal-like AlphaFold2 structures.

The most important free parameter in this module is the lag time, Δ*t*. SPIB identifies the optimal latent space that retains information necessary to predict the system's state label after Δ*t*. Shorter Δ*t* values capture faster motions better and typically produce more states, whereas longer Δ*t* values result in fewer states. In all our test systems and previous work,^41^ we observed consistent latent space representations and a decreasing number of states as Δ*t* increased (Fig. S9†). This indicates that the choice of Δ*t* does not qualitatively affect the neighboring relationships between structures but primarily influences the number of states identified. A more detailed discussion on the selection of parameters can be found in the ESI.†

## Conflicts of interest

There are no conflicts to declare.

## Supplementary Material

DD-004-D5DD00201J-s001

## Data Availability

The code of the 
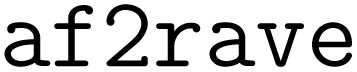
 package is available on GitHub at https://github.com/tiwarylab/af2rave. All other scripts and datasets are available at Mendeley data, at DOI: https://doi.org//10.17632/wz6dtrykj4.1. This includes: (1) the structures generated by AF2 and the config. file used to generate them. (2) The SPIB models trained and training scripts. (3) TICA models and training scripts. (4) Reference structures. (5) Miscellaneous scripts. The millisecond long MD trajectory is available at https://covid.molssi.org/simulations/.
