# Supplementary material for: af2rave: protein ensemble generation with physics-based sampling
Source: Digit Discov. 2025 Jul 4;4(8):2052–61. doi: 10.1039/d5dd00201j (PMC12226971; doi:10.1039/d5dd00201j)
Supplement: DD-004-D5DD00201J-s001 [file DD-004-D5DD00201J-s001.pdf]

Supplementary Information (SI) for Digital Discovery.
This journal is © The Royal Society of Chemistry 2025

Supplementary Information for

af2rave: Protein Ensemble Generation with Physics-Based Sampling

Da Teng, Vanessa J. Meraz, Akashnathan Aranganathan,
Xinyu Gu, and Pratyush Tiwary*

Contents

1 Supplementary Methods **1**
1.1 Hypothesis generation and feature selection . . . 1
1.1.1 ADK . . . 1
1.1.2 DDR1 . . . 2
1.1.3 RBD with full MSA . . . 3
1.1.4 RBD with reduced MSA . . . 4
1.1.5 Discussions on choice of parameters . . . 6
1.2 Simulation details . . . 6
1.3 State Predicted Information Bottleneck (SPIB) . . . 7
1.4 Projections . . . 7
1.4.1 ADK . . . 7
1.4.2 DDR1 . . . 7
1.4.3 RBD: Millisecond-long reference MD and TICA projection . . . 7

2 Latent space generated by SPIB **8**
2.1 ADK . . . 8
2.2 DDR1 . . . 9

1 Supplementary Methods

1.1 Hypothesis generation and feature selection

1.1.1 ADK

For ADK, we generated with 128 seeds at MSA depth 8:16 (640 structures), 128 seeds at MSA depth 16:32 (640 structures), and 5 seeds at full MSA (depth 512:5120, 25 structures). These structures together provided good variety.

Using the best pLDDT prediction from the full MSA run as the reference structure, all 1305 structures had a C_α RMSD of < 6.0 Å (Figure S1). Given this low RMSD, all structures were

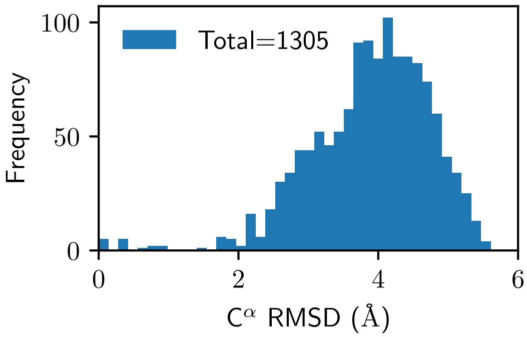

Figure S1: C_α RMSD of generated ADK structures to reference

retained. We used all pairwise distances between the core domain and the NMP domain (residues 30–59) and between the core domain and the LID domain (residues 122–159) as the initial CVs. The core domain was defined as anywhere other than the NMP and LID domain. From each group, the top 200 distances with the highest coefficient of variance were selected, resulting in a total of 400 pairwise distances. The structures were clustered in this 400-dimensional space using regular-space clustering, with a distance threshold of 3 Å. Ultimately, 7 structures were selected, and MD simulations were run for 100 ns from each.

1.1.2 DDR1

For DDR1, only MSA 8:16 was used in the work. As reported in a previous work, this depth is the best for capturing large, potentially slow movement of the Aloop. [3] We used 128 seeds with 5 models to generate a total of 640 structures.

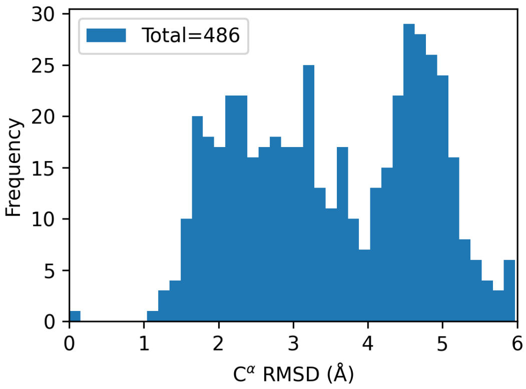

Figure S2: C_α RMSD of generated DDR1 structures with a 6Å filter

The structures were passed through an RMSD filter. The structure with the best pLDDT in the prediction was used as the reference. A filter of 6 Å RMSD was applied and a total of 486 structures were kept. Pairwise distances between the following motifs were used as the initial CVs:

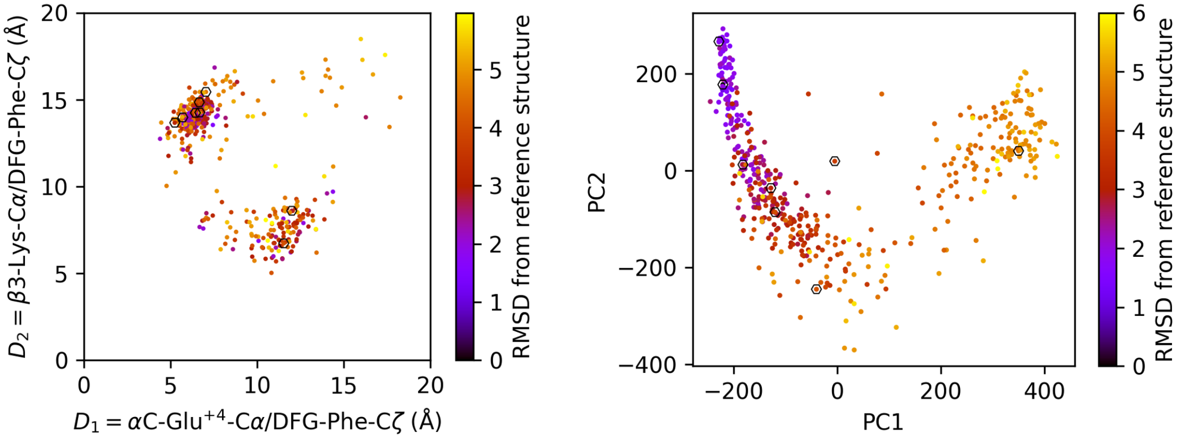

Figure S3: Regular space clustering onto Dunbrack space(left) and PCA space (right)

- Any CA between residue 53 to 82 and 163 to 225
- The CB and CG atom for DFG-Asp(186), CZ and CG for DFG-Phe(186), and O for DFG-Gly(187)
- CD for Glu73
- CB CZ NZ for Lys56, and N for Arg171 (potential salt bridge).

The CA atoms were picked from nearby domains, and those non-CA atoms were picked from reference 7. These 102 atoms yielded 5151 pairwise distances. The top 200 were picked for structural clustering. Regular space clustering was done with a 5.0 Å distance threshold and 7 cluster centers were identified. MD simulations were run for 100 ns for each of these cluster centers.

1.1.3 RBD with full MSA

The case of RBD is special as very few homology sequences are available for this COVID protein. Only 142 sequences were available in the MSA due to its novelty.

The work we reported in the main text utilized full MSA depth which used all the sequences available. The resulting structure had a small RMSD distribution (mostly $<$ 4 Å) (Figure S4), and all structures were kept. For comparison, the structure RMSD of the 1.8 ms long reference MD was shown in Figure S5 where most of the distribution has a RMSD $<$ 4.5Å. The CVs were chosen as the top 200 pairwise distances between all C_α atoms. Regular space clustering was done in this space with a 2.5 Å distance threshold and 21 cluster centers were identified.

When generating the simulation box, four pairs of disulfide bonds were added according to Uniprot entry `P0DTC2 SPIKE_SARS2` [8]. They are between residues (4, 29), (47, 100), (59, 193), and (148, 156).

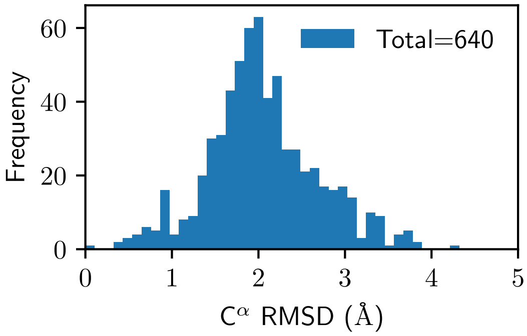

Figure S4: C_{α} RMSD of generated RBD structures to reference

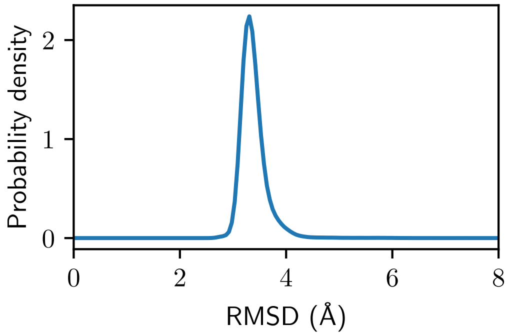

Figure S5: C_{α} RMSD of 1.8 ms-long reference MD simulation

1.1.4 RBD with reduced MSA

In the previous subsection we covered the simulation details of the full MSA RBD as reported in the main text. We also made an attempt of RBD with the same protocol used in ADK with reduced MSA, and in this subsection we will discuss the results of this attempt.

First, with reduced MSA at depth 8:16 and 16:32, the RMSD of the structures compared to experimental structure became much higher. Long MD mostly have RMSD < 4.5 Å (Figure S5) and so is our full MSA calculation (Figure S5). But reduced MSA gave wildly different structures with RMSD mostly in the $2 - 10$ Å region. (Figure S6). We used a cutoff of 5 Å as the first filter. The 8 regular space clustering centers distribute evenly in the PCA space.(Figure S7).

Despite using a modestly small RMSD cutoff similar to what we used for the full MSA one, these structures still deviate significantly from the region where the long MD trajectory lives. After the MD simulation and SPIB protocol, we can see the 8 trajectories do not overlap and form 8 basins with 8 states. In Figure S8, the upper four panels show the trajectories projected in this latent space. The lower four panels show the free energy calculated from unbiased trajectories. This suggests that the structures in the 8 trajectories are so dissimilar that no overlap can be found among them. We also projected the 1.8 ms-long reference MD to this latent space, and the entirety of the trajectory falls into the brown basin.

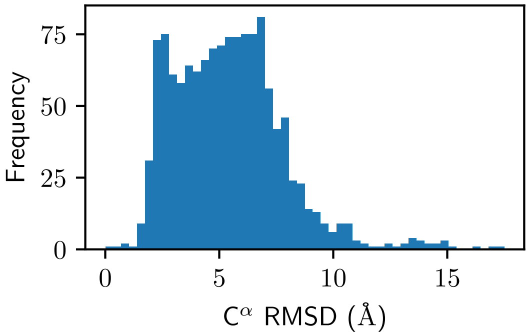

Figure S6: Cα RMSD of reduced MSA structures of RBD, compared to experimental structure

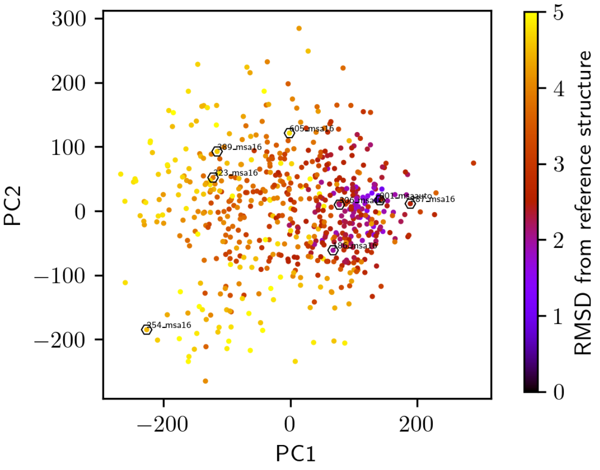

Figure S7: Regular space clustering with reduced MSA RBD

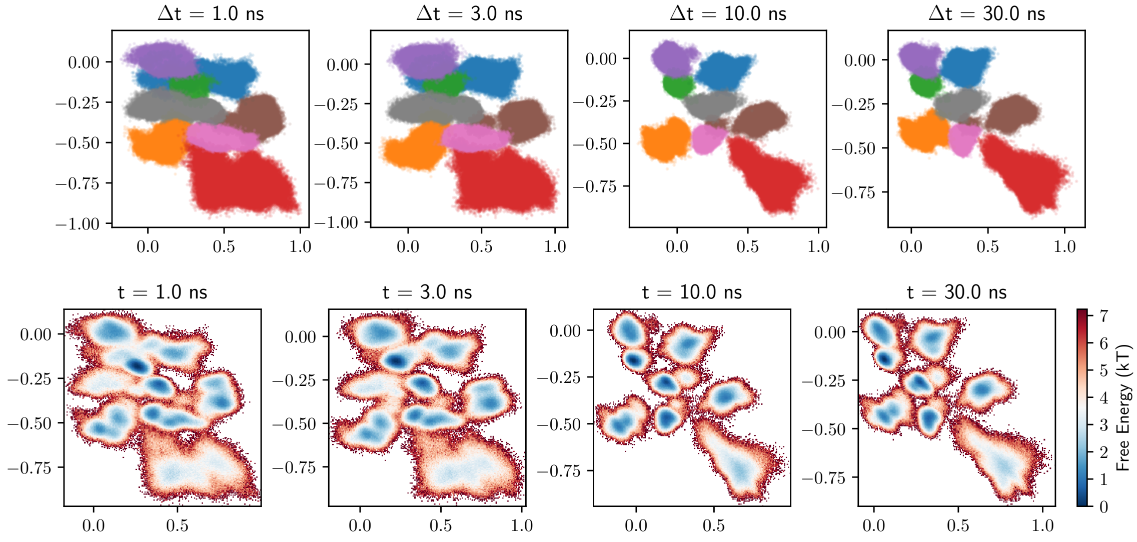

Figure S8: Results from reduced MSA SPIB

With inappropriately generated initial structures, the simulation trajectory can be far away from the experimental structure. They likely still represent some free energy local minima, but are not relevant to the structure features explored in the long MD. The long MD starts with the same initial structure but different initial velocity. It's likely to explore all the regions close to the reduced MSA structures.

1.1.5 Discussions on choice of parameters

In the feature selection module, four important parameters control the output of this section.

MSA depth This has been discussed in the main text about its function in controlling the degrees of diversity the user desires.

RMSD filter In the DDR1 case, we have seen that many structures with high RMSD are often folded incorrectly or very irrelevant to those features we are looking at. Other than RMSD filters, the users are free to use any other filters they see fit.

Number of pairwise distances The third parameter is the selection used to compute pairwise distance. This is where biological prior knowledge can help identify the most important regions to look at. In the case of ADK, we used the distances between the core domain and the other two domains because we know this is the conformation change we are looking for. The same is true for the DDR1 case, where the DFG motif and the activation loop are highlighted. The case in RBD is where we know the least about its conformational diversity, so we used all C_α atoms.

Regular space clustering threshold Often, one wants to keep as many features as possible so as not to miss anything important, but this can lead to a very high dimensional space to do clustering in. Euclidean distance used in regular space clustering can be useless in very high dimensional space. We really liked our choice of 200 CVs in the DDR1 case. On the other hand, the threshold in the regular space clustering mostly controls the number of cluster centers picked. Often, when sampling power is sufficient, the user is encouraged to pick a smaller threshold to get more cluster centers. When computation power is limited, a smaller number is also good enough, but some representative structures might be missed.

1.2 Simulation details

These simulation details were built in as the default parameters in the `af2rave` package.

The simulation box was created with OpenMM.[2] PDBFixer module was used to add hydrogens and missing atoms. Then, a simulation box is created with 10 Å water padding on each side, residues are protonated as in pH= 7 case, and sodium chloride was added to neutralize the box.

The MD simulations were performed using a Langevin integrator with a 2 fs timestep. The temperature was set to 310 K, and the friction coefficient was set to 1 ps^{-1}. A barostat was applied to maintain a pressure of 1 atm. Nonbonded interactions were calculated using a 10 Å cutoff, and long-range electrostatics were treated with the Particle Mesh Ewald method.

ADK and DDR1 The system was simulated with the CHARMM36m force field, and each cluster center was run for 100 ns.

RBD To ensure consistency with the long MD result, which used the AMBER14SB force field, we used the same force field as well. Each cluster center was run for 50 ns.

1.3 State Predicted Information Bottleneck (SPIB)

ADK and RBD Systems were run with default SPIB values and learning rate scheduler gamma at 0.95.

DDR1 Hyperparameter tuning was done in the case of DDR1 with the following changes:

- `neuron_num2 = 256`
- `beta = 1e-3`

1.4 Projections

1.4.1 ADK

The LID and NMP angles were defined following Supplementary Reference 1. Specifically, the angles consist of three centers, each defined by the centers of geometry of the backbone and C_β atoms.

NMP angle θ(NMP) is formed by residues 115–125 (CORE/LID), 90–100 (CORE), and 35–55 (NMP).

LID angle θ(LID), is defined between residues 179–185 (CORE), 115–125 (CORE/hinge/LID), and 125–153 (LID).

1.4.2 DDR1

The salt bridge distance was defined as the distance between the C_β between R191 and D110. The Dunbrack distance and how DFG label distances were assigned was defined as in supplementary reference 6.

1.4.3 RBD: Millisecond-long reference MD and TICA projection

The reference trajectory was downloaded from the MolSSI website at `https://covid.molssi.org/simulations/`. It contains 3000 clones of the same simulation setup, with a total simulation time of roughly 1.8 ms and was saved every 0.5 ns. The sines and cosines of the backbone torsion angles were taken as the CVs and a TICA model was trained with the `deeptime` package.[9, 5] The PMF in the main text Figure 4 was generated with kernel density estimation with a hyperbolic (epanechnikov) kernel and a bandwidth of 0.15.

2 Latent space generated by SPIB

2.1 ADK

Here, we show the four latent spaces with different Δt as generated by SPIB in the case of ADK. The aim is to show the consistency of the latent space across different Δt values.

Figure S9 shows the projection of the MD trajectory to latent space. Each color is from one of the 7 trajectories. Figure S10 shows the state label assigned to the latent space, and Figure S11 is a histogram based free energy from the unbiased MD simulation.

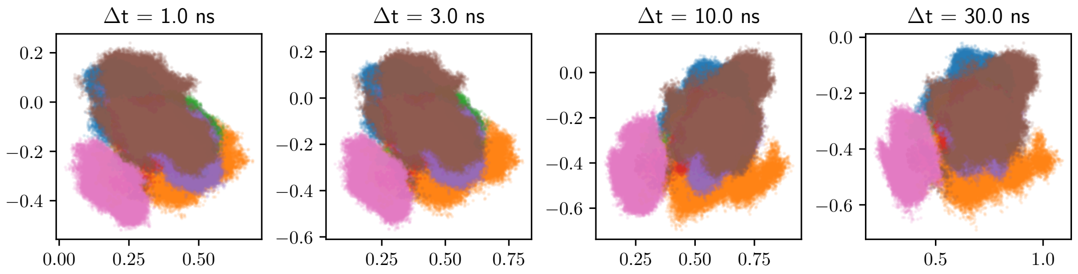

Figure S9: Short MD trajectory of ADK in latent space

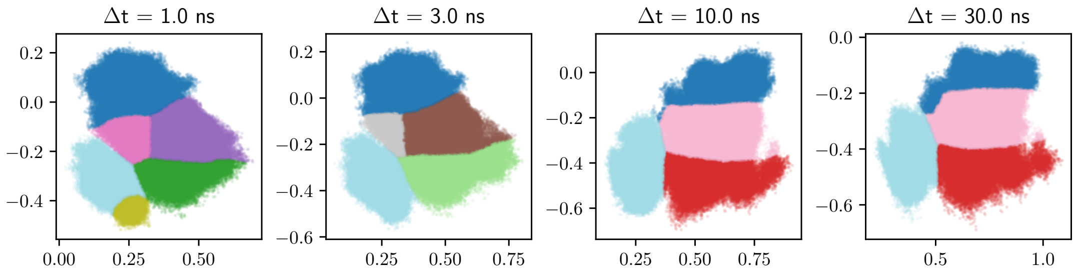

Figure S10: State label designations for ADK MD simulation

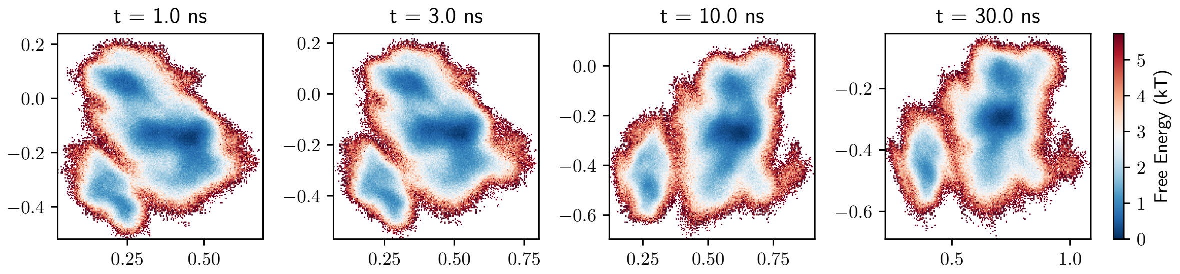

Figure S11: Free energy from ADK unbiased MD in latent space

When tuning the hyper-parameter Δt, we observe that while the number of states decreases (Figure 3F), the relative positions of the trajectories in the latent space remain unchanged. This

indicates that the state label designations at different Δt values differ only in their level of granularity. The lag time can therefore be chosen just to control the number of output states without affecting the overall embedding.

2.2 DDR1

In the DDR1 case, we can also conclude that the latent space is consistent, as with the other cases. We also demonstrate the correlation between latent variables and both DFG labels and salt bridge distances.

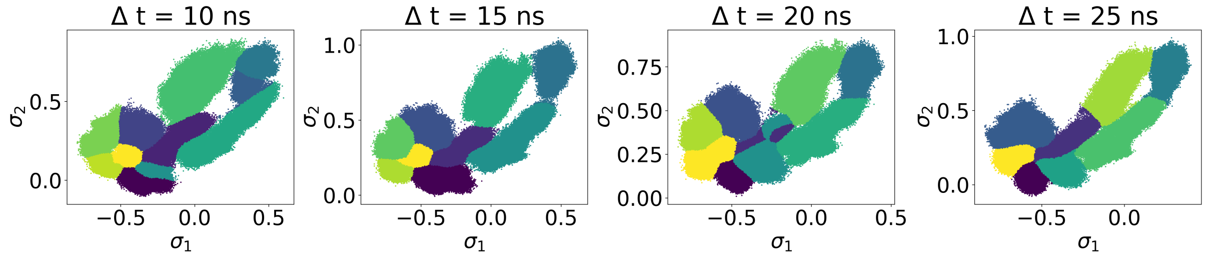

Figure S12: State labels for DDR1 unbiased MD.

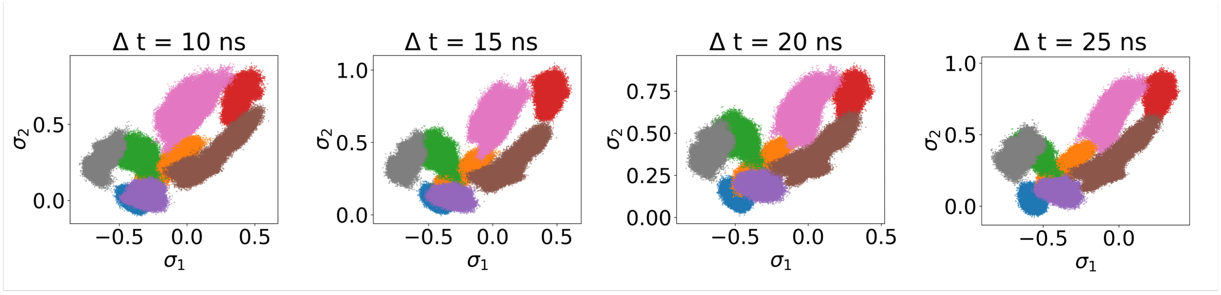

Figure S13: DDR1 unbiased MD in latent space.

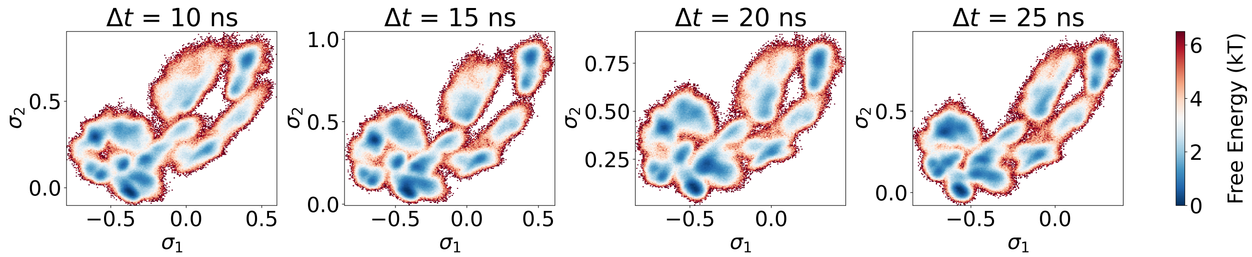

Figure S14: DDR1 Free energy from unbiased MD in latent space.

In the main text, we have shown that across different states we have distinct properties for the DFG label and the salt bridge distance (Figure 2C, D). This is a useful feature when `af2rave` is used for structure generation, but for enhanced sampling or structural projection purposes, it is important that the latent variables can be interpretable to some degree.

Figure S15 shows several projections in latent space at a lag time of $\Delta t = 25$ ns. To instill physical meaning to the latent space, we project the salt bridge and Dunbrack distances onto it. These plots reveal a clear distinction in the latent space as a function of distance.

Along the lower-left to upper-right diagonal, the salt bridge distance increases, indicating a transition of the A-loop from the folded to the extended state. The other diagonal shows a decrease in Dunbrack d_1 and an increase in d_2. Since the two diagonals of the Dunbrack space correspond to DFG-in and DFG-out states, respectively, this diagonal represents the DFG label.

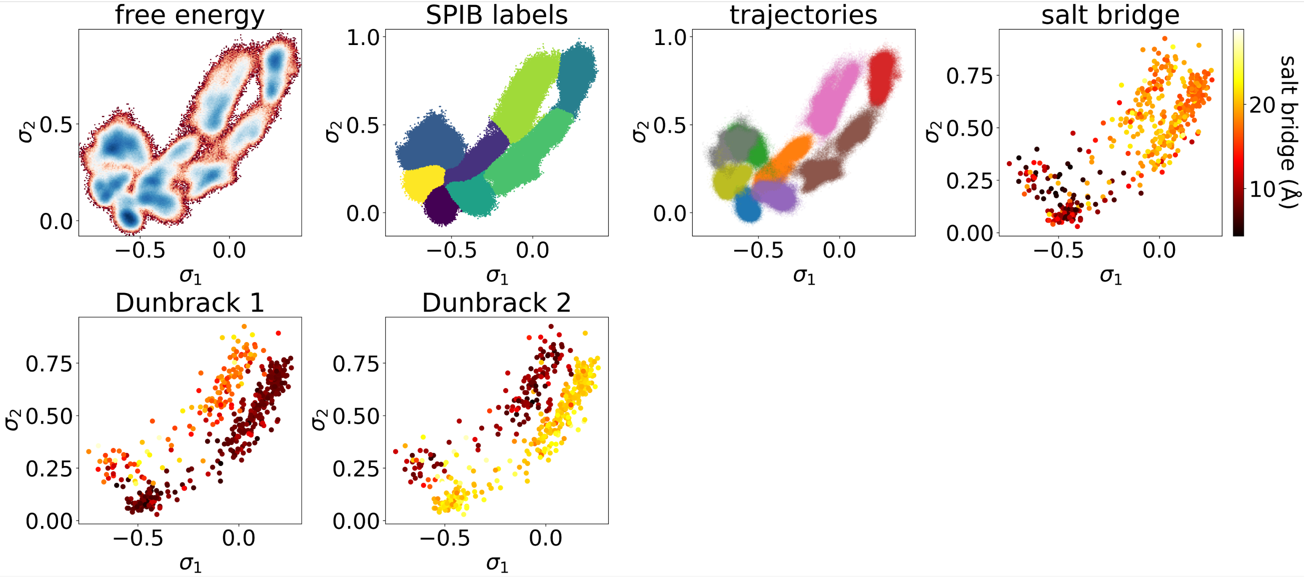

Figure S15: Projections of free energy, state labels, trajectories, the salt bridge, and Dunbrack distances onto latent space for lag time $\Delta t = 25$ ns. For Dunbrack distance, the darker the color the smaller the value.

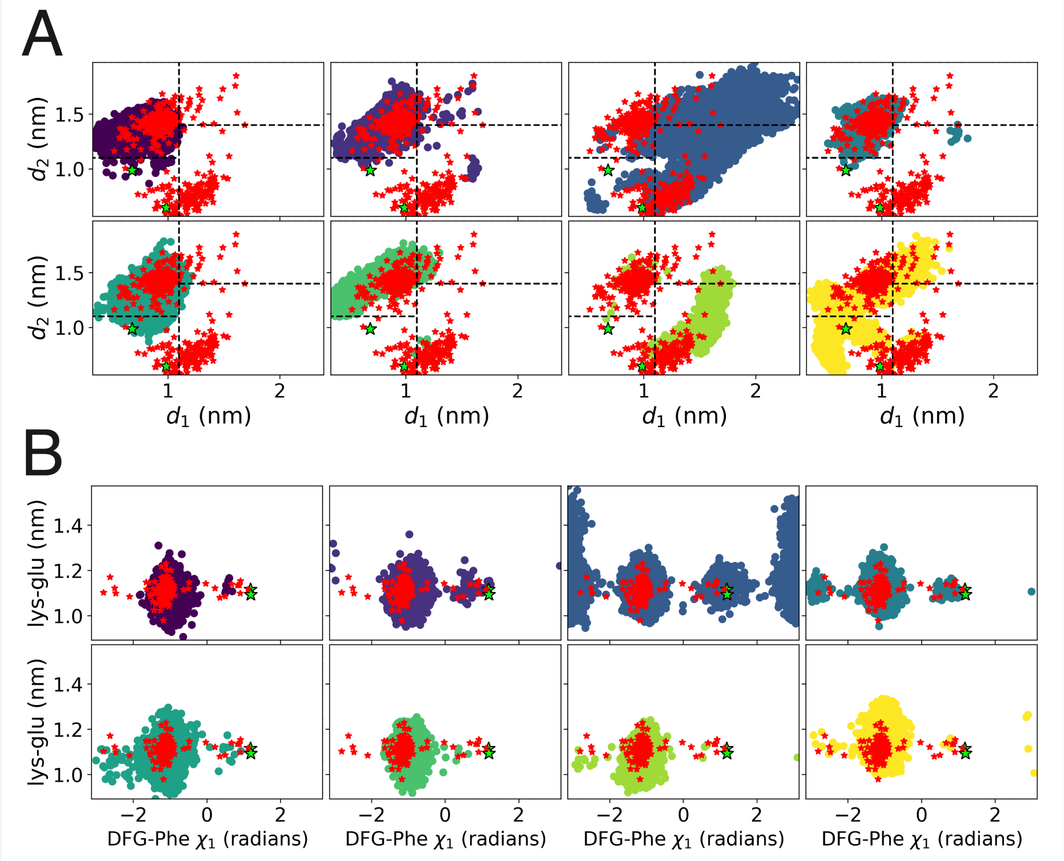

Figure S16: DDR1 SPIB states plotted with respect to (A) Dunbrack distances and (B) the DFG-Phe χ_1 angle against the Lys57-Glu74 distance. Overlaid in red stars are AF2 rMSA results, green stars represent *holo* structures (PDB entries: 6BSD, 6BRJ) [4].

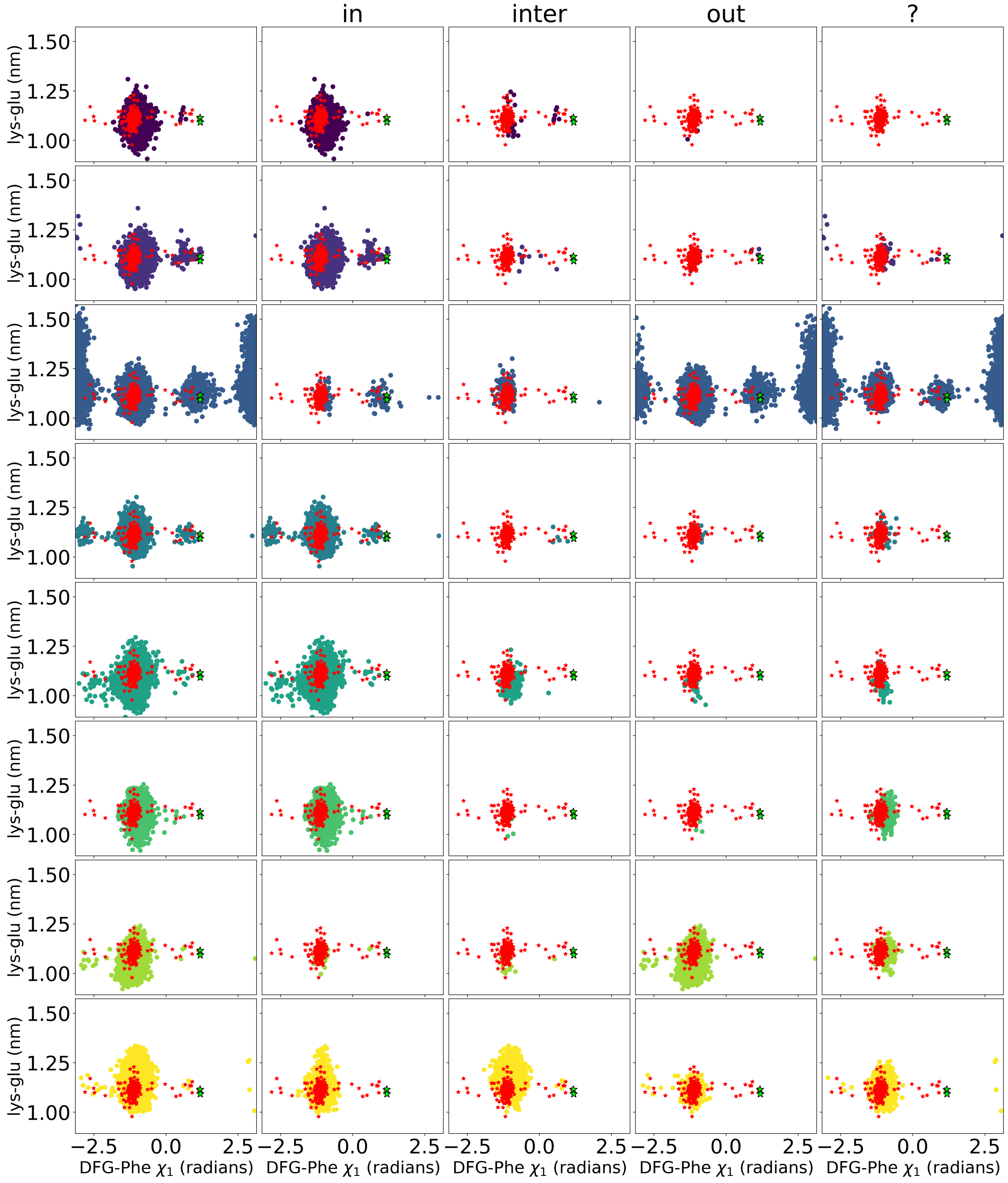

Figure S17: DFG-Phe χ_1 plotted against the Lys57-Glu74 distance. Each row represents a corresponding SPIB state. The first column represents all trajectory data belonging to its respective state. The following columns denote the DFG conformation of in, inter, out, and unassigned (?). Overlaid in red stars are AF2 rMSA results, green stars represent *holo* structures (PDB entries: 6BSD, 6BRJ) [4].

References

[1] Oliver Beckstein, Elizabeth J. Denning, Juan R. Perilla, and Thomas B. Woolf. Zipping and Unzipping of Adenylate Kinase: Atomistic Insights into the Ensemble of Open $\leftrightarrow$ Closed Transitions. *Journal of Molecular Biology*, 394(1):160–176, November 2009.

[2] Peter Eastman, Raimondas Galvelis, Raúl P. Peláez, Charlles R. A. Abreu, Stephen E. Farr, Emilio Gallicchio, Anton Gorenko, Michael M. Henry, Frank Hu, Jing Huang, Andreas Krämer, Julien Michel, Joshua A. Mitchell, Vijay S. Pande, João PGLM Rodrigues, Jaime Rodriguez-Guerra, Andrew C. Simmonett, Sukrit Singh, Jason Swails, Philip Turner, Yuanqing Wang, Ivy Zhang, John D. Chodera, Gianni De Fabritiis, and Thomas E. Markland. OpenMM 8: Molecular Dynamics Simulation with Machine Learning Potentials. *The Journal of Physical Chemistry B*, 128(1):109–116, January 2024.

[3] Xinyu Gu, Akashnathan Aranganathan, and Pratyush Tiwary. Empowering AlphaFold2 for protein conformation selective drug discovery with AlphaFold2-RAVE. *eLife*, 13, August 2024.

[4] Sonya M. Hanson, George Georghiou, Manish K. Thakur, W. Todd Miller, Joshua S. Rest, John D. Chodera, and Markus A. Seeliger. What makes a kinase promiscuous for inhibitors? *Cell chemical biology*, 26(3):390–399.e5, March 2019.

[5] Moritz Hoffmann, Martin Scherer, Tim Hempel, Andreas Mardt, Brian de Silva, Brooke E Husic, Stefan Klus, Hao Wu, Nathan Kutz, Steven L Brunton, and Frank Noé. Deeptime: A Python library for machine learning dynamical models from time series data. *Machine Learning: Science and Technology*, 3(1):015009, December 2021.

[6] Vivek Modi and Roland L. Dunbrack. Defining a new nomenclature for the structures of active and inactive kinases. *Proceedings of the National Academy of Sciences*, 116(14):6818–6827, April 2019.

[7] Bodhi P. Vani, Akashnathan Aranganathan, and Pratyush Tiwary. Exploring Kinase Asp-Phe-Gly (DFG) Loop Conformational Stability with AlphaFold2-RAVE. *Journal of Chemical Information and Modeling*, 64(7):2789–2797, April 2024.

[8] Alexandra C. Walls, Young-Jun Park, M. Alejandra Tortorici, Abigail Wall, Andrew T. McGuire, and David Veesler. Structure, Function, and Antigenicity of the SARS-CoV-2 Spike Glycoprotein. *Cell*, 181(2):281–292.e6, April 2020.

[9] Shuxin Zheng, Jiyan He, Chang Liu, Yu Shi, Ziheng Lu, Weitao Feng, Fusong Ju, Jiaxi Wang, Jianwei Zhu, Yaosen Min, He Zhang, Shidi Tang, Hongxia Hao, Peiran Jin, Chi Chen, Frank Noé, Haiguang Liu, and Tie-Yan Liu. Predicting equilibrium distributions for molecular systems with deep learning. *Nature Machine Intelligence*, 6(5):558–567, May 2024.
